# The role of oxidised self-lipids and alveolar macrophage CD1b expression in COPD

**DOI:** 10.1038/s41598-021-82481-0

**Published:** 2021-02-18

**Authors:** Miranda P. Ween, Jake B. White, Hai B. Tran, Violet Mukaro, Charles Jones, Matthew Macowan, Gregory Hodge, Paul J. Trim, Marten F. Snel, Sandra J. Hodge

**Affiliations:** 1 Department of Thoracic Medicine, Royal Adelaide Hospital, Adelaide, Australia; 2 School of Medicine, Faculty of Health Sciences, University of Adelaide, Adelaide, Australia; 3 Proteomics, Metabolomics and MS Imaging Core Facility, South Australian Health and Medical Research Institute (SAHMRI), Adelaide, Australia; 4 Vascular Research Centre, Lifelong Health Theme, South Australian Health and Medical Research Institute (SAHMRI), Adelaide, Australia; 5 Department of Critical Care, Melbourne Medical School, University of Melbourne, Melbourne, Australia

**Keywords:** Mechanisms of disease, Monocytes and macrophages, Antigen-presenting cells

## Abstract

In chronic obstructive pulmonary disease (COPD) apoptotic bronchial epithelial cells are increased, and their phagocytosis by alveolar macrophages (AM) is decreased alongside bacterial phagocytosis. Epithelial cellular lipids, including those exposed on uncleared apoptotic bodies, can become oxidized, and may be recognized and presented as non-self by antigen presenting cells. CD1b is a lipid-presenting protein, previously only described in dendritic cells. We investigated whether CD1b is upregulated in COPD AM, and whether lipid oxidation products are found in the airways of cigarette smoke (CS) exposed mice. We also characterise CD1b for the first time in a range of macrophages and assess CD1b expression and phagocytic function in response to oxidised lipid. Bronchoalveolar lavage and exhaled breath condensate were collected from never-smoker, current-smoker, and COPD patients and AM CD1b expression and airway 8-isoprostane levels assessed. Malondialdehyde was measured in CS-exposed mouse airways by confocal/immunofluorescence. Oxidation of lipids produced from CS-exposed 16HBE14o- (HBE) bronchial epithelial cells was assessed by spectrophotometry and changes in lipid classes assessed by mass spectrometry. 16HBE cell toxicity was measured by flow cytometry as was phagocytosis, CD1b expression, HLA class I/II, and mannose receptor (MR) in monocyte derived macrophages (MDM). AM CD1b was significantly increased in COPD smokers (4.5 fold), COPD ex-smokers (4.3 fold), and smokers (3.9 fold), and AM CD1b significantly correlated with disease severity (FEV_1_) and smoking pack years. Airway 8-isoprostane also increased in smokers and COPD smokers and ex-smokers. Malondialdehyde was significantly increased in the bronchial epithelium of CS-exposed mice (MFI of 18.18 vs 23.50 for control). Oxidised lipid was produced from CS-exposed bronchial epithelial cells (9.8-fold of control) and showed a different overall lipid makeup to that of control total cellular lipid. This oxidised epithelial lipid significantly upregulated MDM CD1b, caused bronchial epithelial cell toxicity, and reduced MDM phagocytic capacity and MR in a dose dependent manner. Increased levels of oxidised lipids in the airways of COPD patients may be responsible for reduced phagocytosis and may become a self-antigen to be presented by CD1b on macrophages to perpetuate disease progression despite smoking cessation.

## Introduction

Chronic obstructive pulmonary disease (COPD) is incurable and the third leading cause of death world-wide. Identification of new therapeutic targets thus presents an urgent need for health services and a challenging task for translational researchers^1^. The chronic inflammation and the wide range of disease severity seen in patients with identical smoking histories suggest the presence of a self-maintaining pathogenic process although the precise mechanisms for this are unknown. The discovery and increasing number of reports of the presence of self-antigens in COPD patients (even in those with very mild disease and those who have ceased smoking)^2–6^ and other chronic lung disease including Interstitial Lung Disease^7^ and severe eosinophilic asthma^8^ has led to a great deal of debate and interest in the international respiratory community. COPD treatments are rapidly evolving, supported by well-designed clinical trials; however, no treatments to date have stopped the relentless inflammatory response and disease progression.

We were the first to report significant defects in the ability of alveolar macrophages (AM) to phagocytose apoptotic airway epithelial cells (efferocytosis) in COPD and in response to cigarette smoke^9,10^. This uncleared material can then undergo secondary necrosis and perpetuate chronic inflammation^11^ that continues despite smoking cessation^12^. It is likely that the uncleared apoptotic cells can act as immune modulators, exposing lipids that could be oxidised in the highly oxidative airway environment found in COPD patients^13,14^, and potentially recognised and presented as ‘non-self’ by AM^15^. Increased markers of oxidised lipid have been reported in the airways of COPD patients^16,17^ including ex-smokers^18,19^. Increased inflammation has been shown in mice exposed to oxidised lipids^20,21^, antibodies to oxidised lipids were detected in cigarette smoke-exposed mice^21^, and antibodies against self-antigens in systemic lupus erythematosus (SLE) were shown to be related to increased numbers of uncleared apoptotic cells resulting from defective efferocytosis^22^.

**Figure 1:**
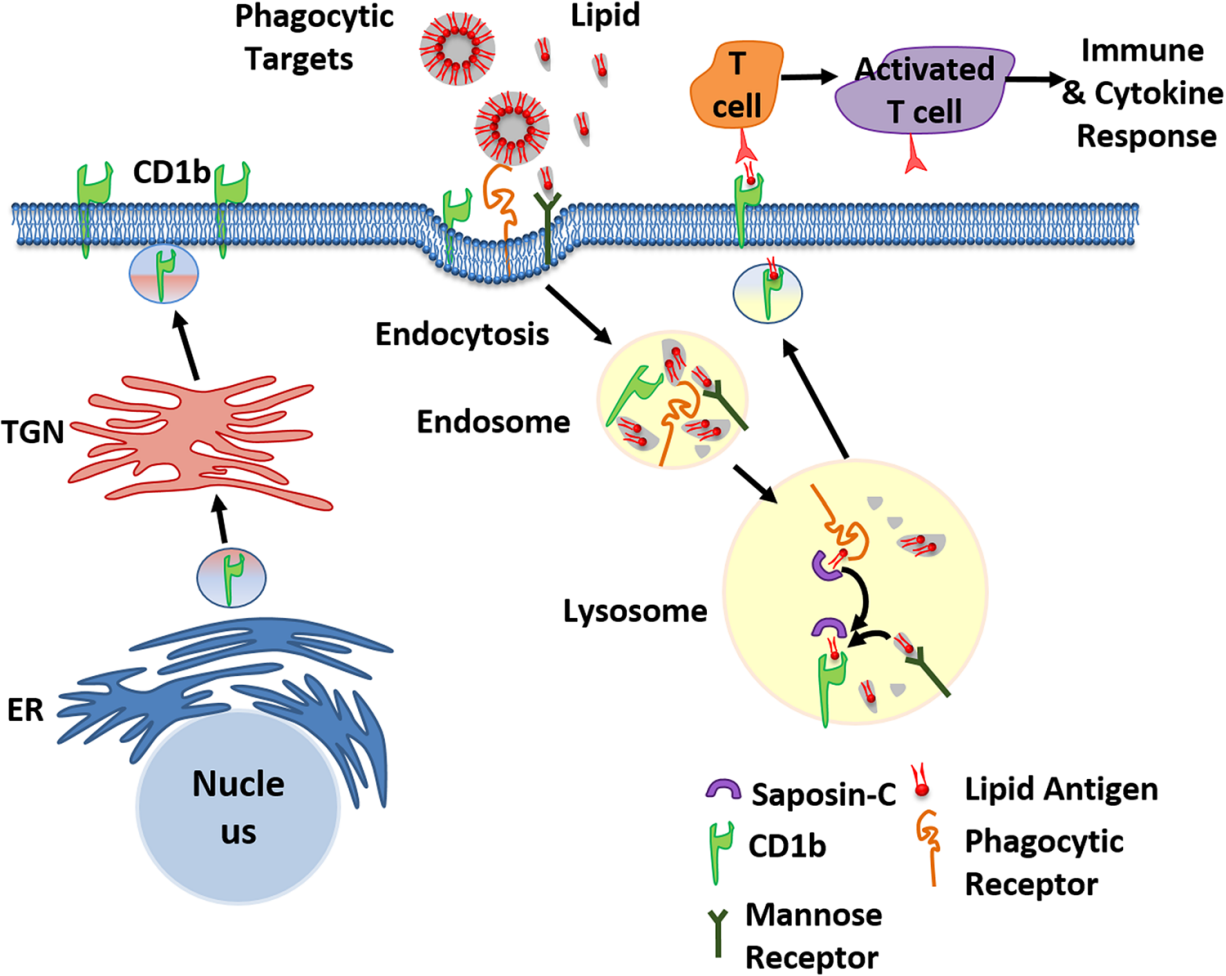
The CD1b lipid presentation pathway. CD1b is produced via the endoplasmic reticulum (ER) and transported to the outer membrane via the trans golgi network (TGN). CD1b is taken back into the cells via phagocytosis and endocytosis along with lipid on phagocytic targets brought in via phagocytic receptors or bound to lipid recognising mannose receptor. Phagocytic targets are broken down in the endosome and lipids loaded onto CD1b in the lysosome via Saposin-C before resorting with bound lipid antigen to the outer membrane where lipids can be presented as an antigen to T cells, resulting in activation and an immune response.

Presentation of protein antigens occurs via well-described traditional presentation pathways such as MHC class I/II. In contrast, little is known about lipid antigen presentation. The CD1 family of glycoproteins have been identified as key to this process^23^. CD1b, in particular has a unique binding groove that allows it to present the greatest range of lipid sizes^23,24^, but has had minimal investigation, in part because it is not expressed in mice^25^. It has so far only been described in dendritic cells^26–29^. CD1b specifically traffics into late endosome/lysosomes where acidic pH and endosomal cofactors including AP1 and AP3 help regulate lipid availability and CD1b complex formation^30,31^. Lipid is taken up into the endosomes and specifically larger lipids may use mannose receptor (MR) to achieve this^32,33^. CD1b may either bind small lipids directly or it is loaded with larger lipids via the sphingolipid activator protein, Saposin C, in the endolysosome^31,34,35^. Antigenic lipids can then be presented to CD1b-restricted T-cells after resorting to the membrane^34,36,37^ (Fig. 1). CD1b so far has been shown to present lipids from mycobacterium tuberculosis bacteria^34^ and also has been shown to bind to several lipids that are exposed during apoptosis^38^.

Given the presence of oxidised lipid markers in the airways of COPD patients, and the links between COPD and autoimmunity, we hypothesised that there would be oxidised lipid in the airway of cigarette smoke-exposed mice and that CD1b would be upregulated in AM from COPD subjects. We further hypothesised that oxidised lipid from bronchial epithelial cells would induce an increase in macrophage CD1b expression and inhibit phagocytic function.

## Methods

### Primary AM and exhaled breath condensate collection

Thirty five never-smoker controls, 23 current smokers, 12 ex-smokers, and 102 COPD subjects were recruited for the study. Fifty COPD subjects were ex-smokers while 52 were current smokers. COPD was diagnosed using the GOLD criteria with clinical correlation or by CT scan. The Royal Adelaide Hospital Human Research Ethics Committee approved the protocol and informed consent was obtained. No identifying information is presented in the manuscript. Never-smoker controls had no history of respiratory or allergic disease and normal spirometry; current smokers had a smoking history of at least 10 pack years; Ex-smokers had ceased smoking for at least 12 months. Correlations between study parameters and demographics, presence of cancer, lung function, smoking status and pack year and bronchoalveolar lavage (BAL) differential leucocyte counts were recorded.

BAL was obtained using flexible bronchoscopy and AM were isolated from BAL fluid (BALF) as previously reported^10^. Exhaled breath condensate (EBC) was collected using a commercial EBC collector as per manufacturer’s instructions (RTube; Respiratory Research, Inc., Charlottesville, VA). Samples were immediately stored at − 80 °C until use.

### Immunofluorescence and confocal microscopy

CD1b intracellular distribution was measured in fixed cytospin preparations from control and COPD AM using immunofluorescence and confocal microscopy as reported^39^. Briefly, cells were permeabilized with 0.1% Triton X100 in PBST for 10 min, blocked for 1 h with serum-free protein blocker (Dako Denmark A/S, Glostrup, Denmark), incubated overnight at 4 °C with rabbit mAb clone EP7251 (Abcam, Cambridge, UK; 1:25) as primary antibody for CD1b, then 45 min at room temperature with secondary antibody, donkey F(ab)2 anti-rabbit IgG-AF594 (Jackson ImmunoResearch, West Grove, PA, USA; 1:200). Imaging was carried out using a LSM confocal system (Carl Zeiss Australia, North Ryde, NSW, Australia).

### 8-Isoprostane measurement

8-Isoprostane levels in BAL and EBC were determined according to the manufacturer's instructions (Cayman Chemicals, Ann Arbor, MI).

### Malondialdehyde in lungs of cigarette smoke-exposed mice

Lung paraffin tissue blocks were archived from a previous mouse model of 6 weeks exposure to cigarette smoke^40^ with protocols approved by the Institute of Medical & Veterinary Science Central Northern Adelaide Health Service animal ethics committee following the Australian National Health and Medical Research Centre “Guidelines to promote the wellbeing of animals used for scientific purposes” and “Australian code for the care and use of animals for scientific purposes”. Sections were cut at 5 μm thickness from 12 mice (six exposed to cigarette smoke (CS) and six control) and were mounted on three tissue arrays and all animals were analysed in the same batch. Immunofluorescence was carried out as previously described^40^. A rabbit polyclonal antibody to malondialdehyde (Abcam ab6463, Cambridge, UK, 1:100) was applied as the primary antibody; a donkey IgG F(ab′)2 fragment conjugated with Alexa Fluor 594 (Jackson ImmunoResearch, West Grove, PA, USA, 1:200) as the secondary. For quantitative immunofluorescence, ten optical fields were captured under 60× objective per animal, MFI was then measured by ImageJ from all available bronchiolar epithelia.

### Preparation of cigarette smoke extract

Cigarette smoke extract was prepared and used at a concentration of 10% as previously reported^41^. Briefly, smoke from 2× reference cigarettes (1R5F, University of Kentucky, KY, USA) was bubbled through 10 mL serum free (to prevent contamination from lipids in FCS) RPMI 1640 media (Life Technologies), supplemented with 12 µg/mL penicillin 16 µg/mL gentamycin (Life Technologies) and l-glutamine (2 mM, Life Technologies) using a vacuum for 5 min per cigarette.

### Preparation of oxidised lipid

The 16HBE14o- (HBE) bronchial epithelial cell line was maintained as previously reported^41^ and regularly tested for mycoplasma infection. For cigarette smoke-induced lipid oxidation, two 1R5F cigarettes with the filter removed were bubbled through a cell suspension then incubated for 2 h at 37 °C, 5% CO_2_ to allow oxidation to occur. Cells were sonicated, lipid extracted with the Folch chloroform/methanol method then dried down to a film, and stored under nitrogen. Oxidation was confirmed by spectrometry at 234 nm in 90% ethanol, which detects conjugated dienes present in oxidised lipid products.

### Sample preparation and LC–MS

Lipid extracts (n = 10) were reconstituted in 990 µL MeCN:IPA (1:1 v/v) briefly vortex mixed, and centrifuged for 15 min *ca* 16,000 × *g*. 100 µL supernatant was transferred to a total recovery vial (Waters Corporation, USA). Mass spectrometric analysis was performed using an Acquity I-Class UPLC system (Waters Corporation, USA) fitted with a 2.1 × 100 mm CSH C18 analytical column heated to 55 °C. Lipids were separated by gradient separation as shown in Table 1. Mobile phase A was 10 mM ammonium formate 60:40 acetonitrile:water with 0.1% formic acid and mobile phase B was 10 mM ammonium formate 90:10 propan-2-ol:acetonitrile with 0.1% formic acid.

**Table 1:** Gradient conditions for LC–MS analysis.

Time (min)	Flow rate (mL/min)	% A	Curve
0	0.4	60	–
2	0.4	57	6
2.1	0.4	50	1
12	0.4	46	6
12.1	0.4	30	1
18	0.4	1	6
20.4	0.4	1	6
20.5	0.4	60	1
22.5	0.4	60	1

Online MS analysis was performed using a Xevo G2-XS Q-ToF (Waters Corporation, Manchester) in positive ion sensitivity mode over the *m/z* range 120–1200.

### Mass spectrometry data analysis

Raw mass spectrometry datafiles were imported into Mzmine2 (version 2.52) for peak picking and chromatogram integration^42^. The function 2 lockmass channel was deleted prior to analysis, as mass correction was applied at acquisition. Batch mode was used to process all datasets in parallel. Masses with intensity higher than 1000 were chosen for mass list generation. Chromatograms for each mass were identified using the previously published ADAP chromatogram builder module^43^. Mass tolerance to generate extracted ion chromatograms (EIC) was set to the larger of 0.007 Da or 1.0 ppm. EICs were deconvoluted with the ADAP wavelets modules using a S/N threshold of 6, minimum feature height of 4000 and coefficient/area threshold of 80. Chromatographic peak elution time was set between 0 and 1.1 min, RT wavelet range was set between 0.001 and 0.500. Deconvoluted, integrated chromatograms were compared to an in-house database based on exact mass, retention time and dominant ion adducts to identify lipid species. An in-house QC sample of pooled plasma was processed in parallel to ensure all matching lipid species were correctly identified. Lipid species were resolved to the combined total carbon chain length and double bond count in all acyl chains. Identified lipid peak areas from smoke and control samples were used to provide qualitative analysis of relative lipid distribution between groups.

### Class based lipid distribution

Distribution of lipids from each class was obtained by finding the total ion area of each class from each sample, and the class signal as a proportion of the total ion area were obtained (Eq. 1). Percentage distribution values from each treatment group were averaged to obtain a qualitative view of the class-based distribution between samples

1 $$Class\;distribution\;per\;sample = \frac{Total\;ion\;area\;of\;class}{{Total\;ion\;area\;of\;all\;identified\;lipids}}$$

### Double bond based lipid distribution

The proportion of lipids with a given number of double bonds across both acyl chains was obtained by finding the average percentage distribution of each double bond count in each sample (Eq. 2). This result was then averaged per treatment group

2 $$Double\;bond\;distribution\;per\;class = \frac{Total\;ion\;area\;of\;double\;bond\;count\;n\;in\;class}{{Total\;ion\;area\;of\;all\;identified\;lipids\;in\;class}}$$

### Monocyte-derived macrophages (MDM)

To provide large cell numbers required for our studies we also employed MDM as we have shown them to be excellent surrogates for AM^44^. Venous blood was collected from ten additional healthy control volunteers into 10 U/mL preservative free sodium heparin (DBL, Sydney, Australia). The Royal Adelaide Hospital Human Research Ethics Committee approved the protocol and informed consent was obtained. MDM were differentiated by culturing monocytes isolated from PBMC by adhesion to plastic for 1 h, washing non-adherent cells and culturing in RPMI 10% FCS and 2 ng/mL GM-CSF for 12 days, with media changed on day 4 and day 8 to create “alveolar-like” macrophages^45^.

### Treatment of MDM with cigarette smoke or oxidised lipids

Lipid was resuspended into serum free media by sonication for 20 cycles on high (Bioruptor Plus, Diagenode NJ, USA). MDM were treated for 24 h at 37 °C, 5% CO_2_ in serum-free media (to limit presence of other lipids, including sphingolipids) with control serum free media, control lipid, 10% cigarette smoke extract, or cigarette smoke-oxidised lipid (0–100 µg/mL).

### Bronchial epithelial cell culture and measurement of cellular toxicity

The 16HBE14o-bronchial epithelial cell line (16HBE) was a generous gift from Dr. Dieter C. Gruenert (University of California, San Francisco, CA). Cells were maintained in MEM medium supplemented with l-glutamine (2 mM), Penicillin (12 µg/mL) and Gentamycin (16 µg/mL) with 10% FCS. 16HBE were seeded at a density of 0.7 × 10^6^ cells/cm^2^ in serum free media for 24 h prior to treatment for 24 h. 16HBE cells were trypsinized and incubated with annexin binding buffer for 10 min (ABB:10 mM HEPES pH 7.4, 150 mM sodium chloride, 5 mM potassium chloride, 1 mM magnesium chloride, 1.8 mM calcium chloride). Cells were incubated with 3 µL Annexin V-APC (BD Biosceinces) and 2 µL of 1 µM Sytox Green nucleic acid stain (Life technologies). Gating strategy and evidence of 16HBE as a suitable model for primary bronchial epithelial cells was recently published^41,46^ and representative staining cytometric plots are provided in Fig. S4.

### Flow cytometric measurement of CD1b/HLA class I/II and mannose receptor

Macrophage expression of CD1b, MHC antigen-presenting molecules class I (HLA-ABC) and class II (HLA-DR) and mannose receptor were measured by flow cytometry and gated as described^47^. All antibodies were conjugated and were purchased from BD Biosciences, San Jose, CA USA except for the majority of CD1b staining which was performed either using a FITC conjugated antibody from BD Biosciences for the comparison of CD1b expression in AM of control, smoker, ex-smoker, and COPD patients, or an unconjugated mouse anti-CD1b and purchased from Santa Cruz Biotechnology, Dallas, Tx, USA and paired with an APC-conjugated secondary antibody from eBiosciences, San Diego, CA, USA for analysis in MDMs. This consisted of an initial step with the unconjugated antibody, a wash, a second step with an anti-mouse-APC secondary antibody, a wash, and then a final step with the HLA-ABC-FITC antibody, a wash and then measurement. Unstained treated macrophages or secondary antibody only stained cells were used for gating controls as appropriate for the staining method and representative plots are provided as Figs. S1 and S3. Assessment was in multicolour panels that were designed to prevent overlap in the emission spectra. Ten thousand events were collected and analysed using FACS DIVA 7.0 and expressed as % positive. Representative staining cytometric plots are provided in Figs. S1 and S3.

### Macrophage phagocytic function

Non-typeable *H. influenzae* (NTHi), prepared as previously described, were applied as phagocytic targets to avoid potential interference of lipids exposed on apoptotic cell targets^47^. We have previously confirmed a strong correlation between macrophage phagocytic function using either bacteria or apoptotic cell targets. Briefly, macrophages were treated for 24 h. pHrodo labelled NTHi were incubated with macrophages for 90mins at 37 °C 5% CO_2_. Non-phagocytosed NTHi were removed, the wells rinsed three times, and the macrophages lifted with trypsin and FCS media to quench. Macrophages were centrifuged at 1100 × *g*, vortexed, washed with the HBSS/HEPES solution before reading 10,000 events on a FACSCanto II flow cytometer (BD Biosciences). Macrophages not exposed to NTHi were used as a gating control and phagocytosis was assessed using FACS DIVA 7.0 (BD Biosciences) and data expressed as the percentage of positive cells. Gating strategies have been previously reported^47^.

### Statistical analysis

Analysis was performed using SPSS software (v25 https://www.ibm.com/au-en/analytics/spss-statistics-software) or Graphpad Prism (v8 https://www.graphpad.com). The Friedmans non-parametric test with the Wilcoxon signed ranks pairwise test were performed for analysis of the MDMs and data are mean ± SEM unless otherwise indicated. Bivariate correlation analyses between CD1b and demographic variables were performed using Spearman Rho correlation tests. Differences between groups of 2-tailed p < 0.05 were considered significant. For mouse MDA analysis, we applied an unpaired T-test, as variance was found to be non-significant. For each outcome, an analysis of variance analysis was performed to investigate whether the presence of cancer had an effect, and both an interaction and main effects models were investigated.

### Ethics approval and consent to participate

The Royal Adelaide Hospital Human Research Ethics Committee approved the protocol and informed consent was obtained from all participants. Animal protocols were approved by the Institute of Medical and Veterinary Science Animal Ethics Committee. All methods were carried out in accordance with relevant guidelines and regulations.

## Results

### Patient demographics

The demographic details of subjects tested are presented in Table 2.

**Table 2:** Demographics of patients who underwent BAL and/or EBC collection.

Patient group	Number	Age	FEV_1_	FEV_1_/FVC
Never-smoker control	35	53 (21–72)	100 (78–134)	84 (66–93)
Smoker	23	52 (24–67)	88 (74–112)	73 (61–87)
Ex-smoker	12	58 (29–74)	101 (85–123)	77 (71–89)
COPD smoker	46	58 (34–91)	68.5 (38–102)	61 (28–76)
COPD ex-smoker	51	68.5 (45–85)	63 (21–89)	58.5 (34–83)

Data represents the median and the min and max of all patients in each group. Not all patients were used for each experiment. FEV_1_ data is presented as percentage of predicted values.

### CD1b expression is increased on AM from COPD patients and smokers

**Figure 2:**
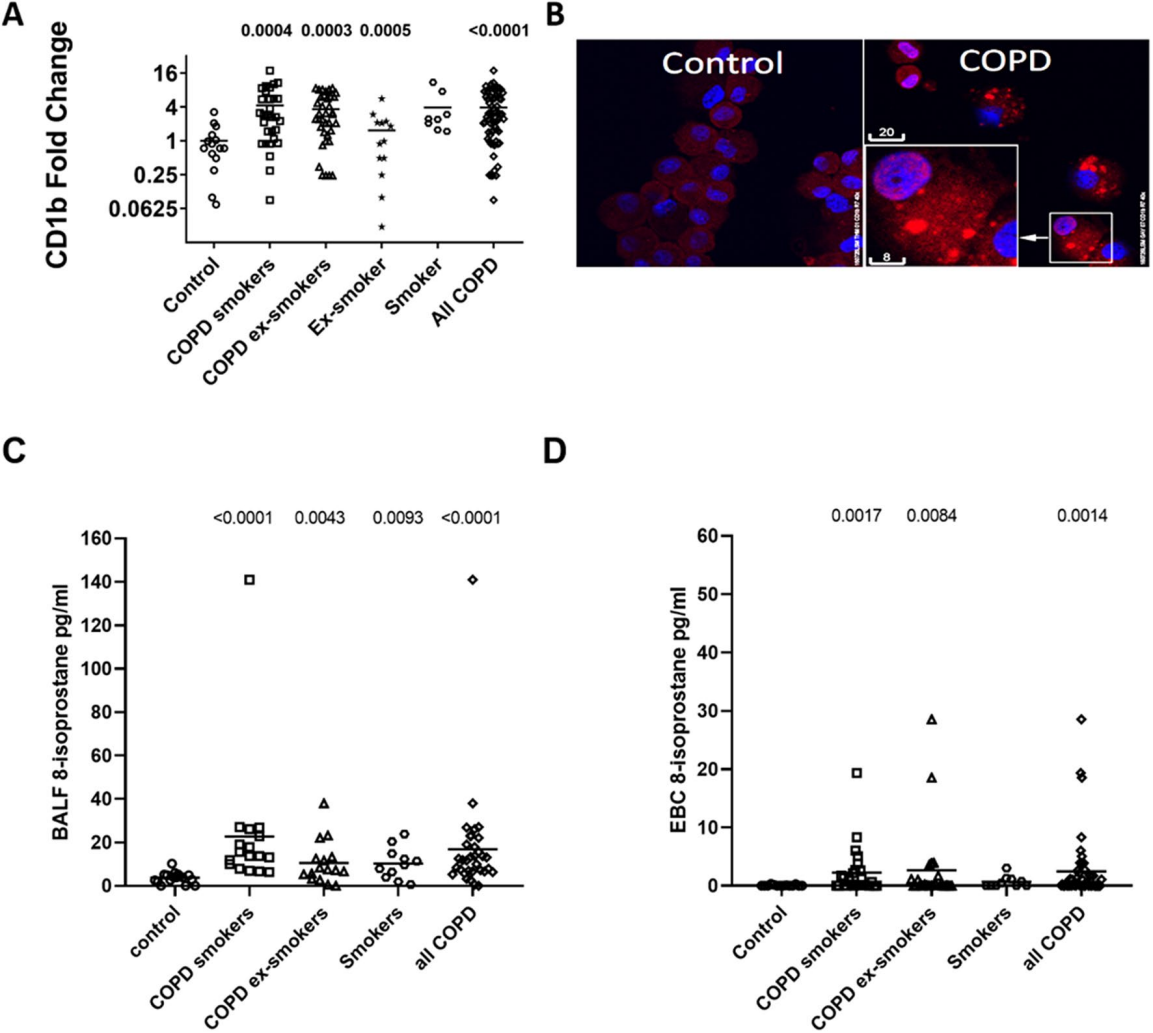
CD1b expression in alveolar macrophages and 8-isporostane in the airways of COPD patients and smoker*s.* (**A**) CD1b expression was assessed by flow cytometry using a FITC-conjugated antibody in alveolar macrophages from never-smoker controls (n = 14), ex-smokers (n = 12), current smokers (n = 7), and current- and ex-smoker COPD subjects (n = 29 and n = 36 respectively). Data presented as fold change relative to mean of never-smoker controls. *p* values vs control provided from Mann Whitney U test. (**B**) Representative confocal images showing increased CD1b (red) in alveolar macrophages from a COPD subject vs. never-smoker control. DAPI (blue); scale bar (μm). 8-isoprostane in (**C**) BALF from never-smoker controls (n = 16), current smokers (n = 11), and current- and ex-smoker COPD subjects (n = 16 and n = 15, respectively) and (**D**) EBC from never-smoker controls (n = 13), current smokers (n = 10), and current- and ex-smoker COPD subjects (n = 24 and n = 23, respectively). Data represents individual values and the mean. Significance from control at *p* < 0.05. Mann Whitney U test.

Using flow cytometry, we showed that both AM and MDM similarly express CD1b (data not shown), confirming the suitability of using MDM as surrogates for AM in experiments where larger numbers of cells were required. Expression of CD1b was significantly increased in AM from COPD subjects (both current- and ex-smokers) and smokers compared with healthy never-smoker controls (Fig. 2A). These findings were confirmed using confocal imaging (Fig. 2B). We further observed increased 8-isoprostane, which is produced non-enzymatically from arachidonic acid during the peroxidation of membrane lipids, in the BAL fluid of smokers, COPD smokers, and COPD ex-smokers (Fig. 2C) and in the EBC of COPD smokers and ex-smokers (Fig. 2D).

### CD1b expression correlates with COPD disease severity and smoking pack year

CD1b expression significantly correlated with smoking pack year history (Spearman Rho correlation coefficient 0.362, p = 0.003) and lung function (FEV_1_) (correlation coefficient − 0.289, p = 0.031). There were no significant correlations between CD1b and age, gender, or any leucocyte subset in BAL (Table 3).

**Table 3:** Correlations between various factors of COPD and CD1b AM expression.

Measure	Spearman coefficient	*p* value
Pack year history	0.362	0.003
FEV_1_	− 0.289	0.031
Age	0.140	0.264
Gender	0.194	0.121
BAL eosinophils	− 0.52	0.712
BAL macrophages	0.116	0.411
BAL neutrophils	− 0.133	0.426

### Cigarette smoke exposure induces lipid peroxidation in mouse bronchiolar epithelia

**Figure 3:**
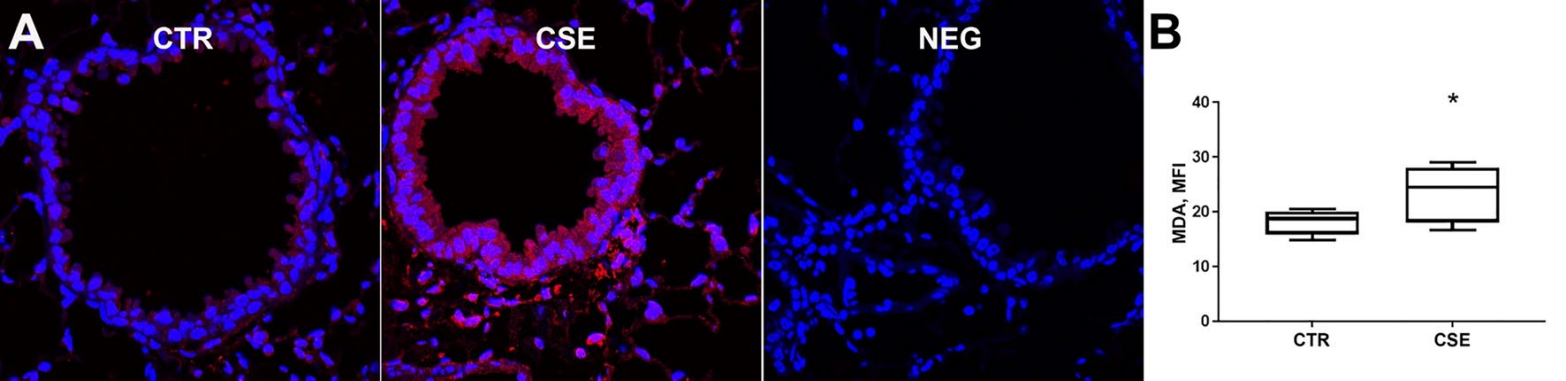
Cigarette smoke significantly increases malondialdehyde in mouse bronchial epithelia. (**A**) Representative immunofluorescence images of malondialdehyde staining in lung paraffin tissue blocks from control mice (CTR) and mice exposed to cigarette smoke for 6 weeks (CSE). (**B**) Box plot of malondialdehyde MFI in bronchial epithelia of CTR and CS-exposed mice (n = 6 per group). *p < 0.05 vs. controls via unpaired t-test.

Malondialdehyde, a product of lipid peroxidation of polyunsaturated fatty acids, was shown to be significantly elevated in bronchiolar epithelium of cigarette smoke-exposed mice compared to non- exposed control mice (mean MFI of 18.18 vs 23.50, *p* = 0.0375, Fig. 3A,B).

### Cigarette smoke extract induces oxidised lipids from bronchial epithelial cells

**Figure 4:**
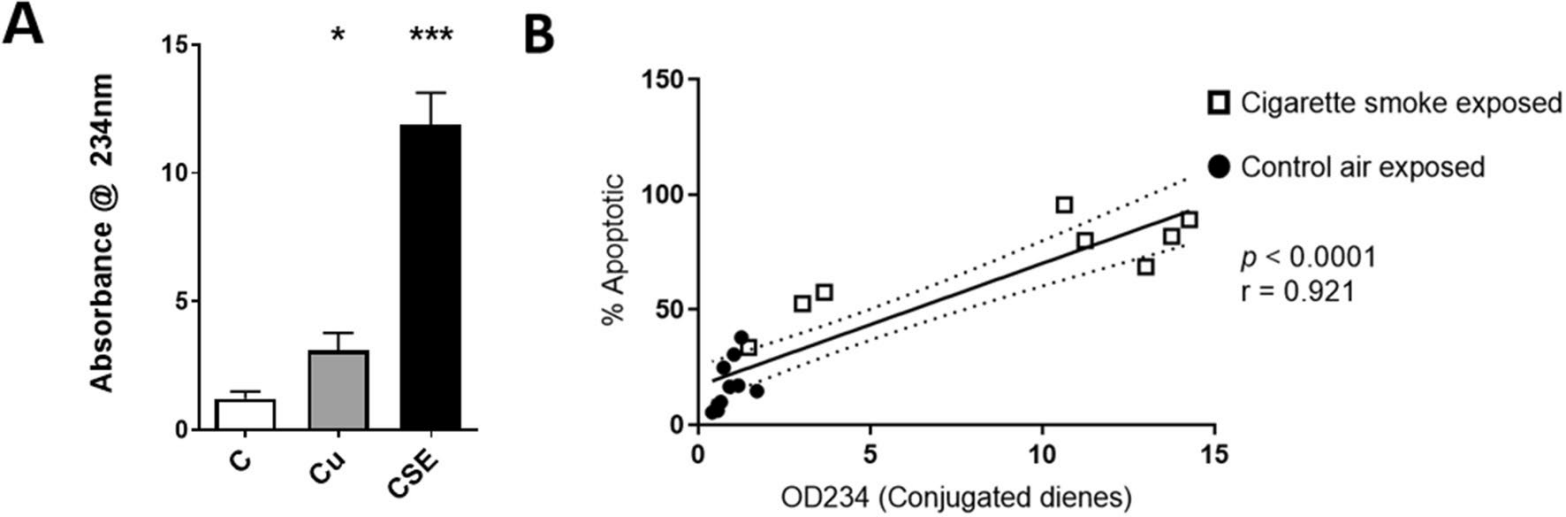
Cigarette smoke extract induces oxidised lipids from bronchial epithelial cells. (**A**) Relative levels of lipid oxidation in CSE-treated HBE cells measured by absorbance at 234 nm (peak absorbance of conjugated dienes); copper (Cu) was used as an oxidation control. Data represents mean ± SEM, n = 6–10, *p < 0.05, ***p < 0.005, Wilcoxon signed ranks test. (**B**) Spearman correlation of lipid oxidation (by OD234 measurement) and percentage of apoptotic cells.

Total lipid extracted from CS-exposed HBE cells showed a 9.8-fold increase in lipid oxidation products vs total lipid from control treated cells confirmed by spectrometry at 234 nm, which detects conjugated dienes present in oxidised lipid products (Fig. 4A). Cu (oxidation stimulant) showed a 2.54-fold greater oxidation than control lipid but far lower oxidation than cellular lipid from CS treated cells. When assessing the rate of apoptosis just before control and CS treated cell lipid extraction, the rate of apoptosis directly correlated with the oxidation status (Fig. 4B).

### Analysis of oxidised vs non-oxidised lipids

**Figure 5:**
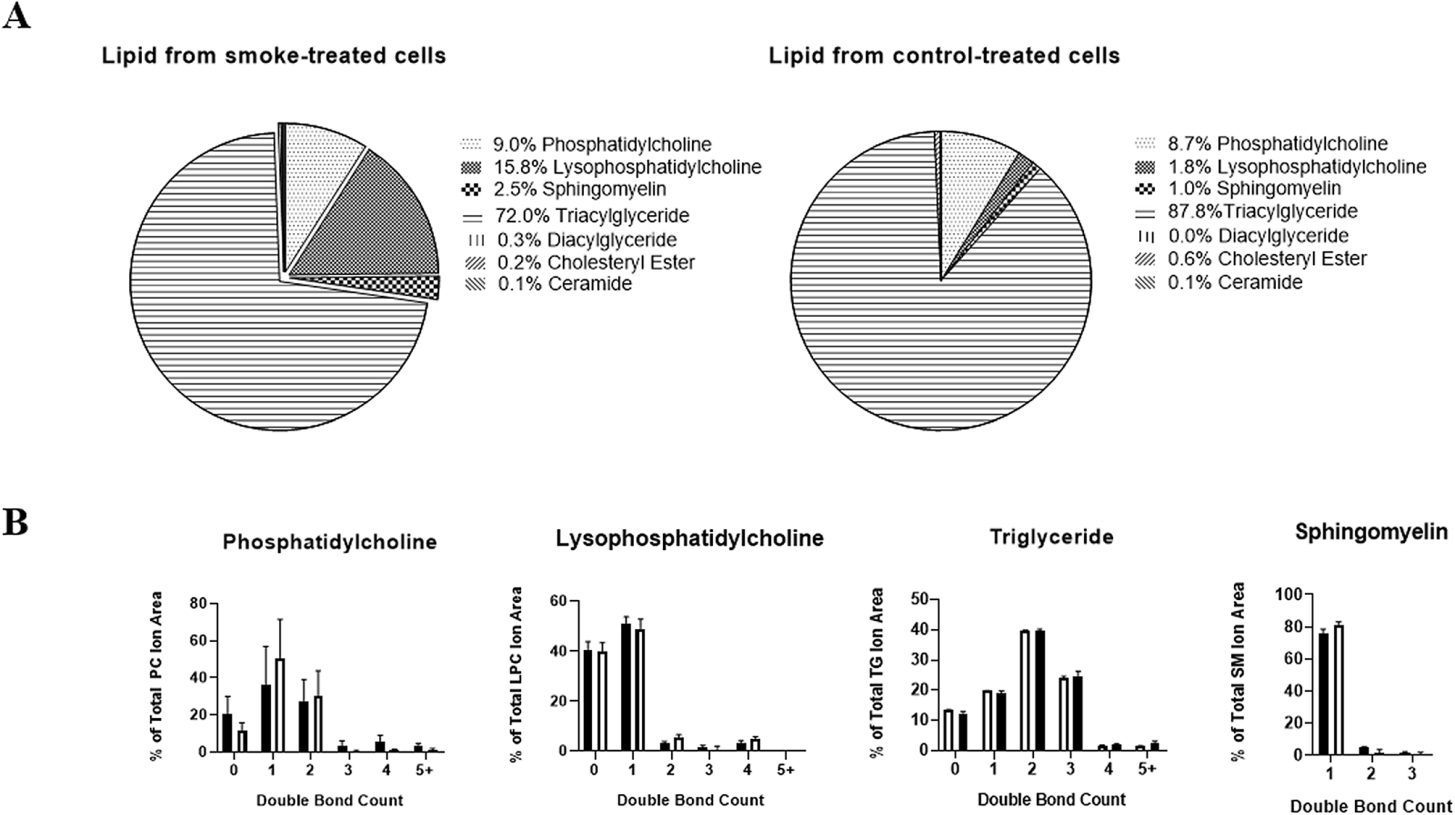
Lipid distribution by class and double bonds within major classes in 16HBE total cellular lipid. (**A**) Distribution of lipids from each class was obtained by finding the total ion area of each class from each sample, and the class signal as a proportion of the total ion area were obtained. Data represents an average of each treatment group to obtain a qualitative view of the class-based distribution between samples. N = 5 samples per treatment group (**B**) The proportion of lipids with a given number of double bonds across both acyl chains was obtained by finding the average percentage distribution of each double bond count in each sample. Data are presented as average per treatment group + SD. N = 5 samples per treatment group. Total lipid extracted from White = control or black = smoke exposed 16HBE cells.

When comparing lipid class changes between total cellular lipid from smoke-exposed and control cells, the smoke-exposed lipids showed relative increases in lysophosphatidylcholine (15.8% vs 1.8% for control) and sphingomyelin species (2.5% vs 1% for control) when compared to controls (Fig. 5A). Triglyceride composition was relatively lower in cellular lipid from smoke-treated cells when compared to controls (72% vs 87.8%). Analysis of naturally occurring lipids (rarely contain conjugated dienes) via LC–MS showed no change in the di-sulfide bonds distribution within classes between control and CS-oxidised lipid samples (Fig. 5B). The standard deviation for all classes was below 5%, except for phosphatidylcholine species, which had higher variation for lipids with 0 (9%/4% SD in smoke/control), 1 (20%/21% SD) or 2 (12%/14% SD) double bonds across both acyl chains. (Fig. 5B).

### CD1b levels are higher in AM and alveolar-like M1 MDM

CD1b levels were observed to be highest in AM and our alveolar-like M1 MDM model with no significant difference between the two cell types. In comparison, CD1b levels on monocytes and M2-like MDM were statistically similar but significantly lower than both AM and alveolar-like M1 MDM (Fig. S2).

### Oxidised epithelial cell lipids increase macrophage CD1b expression

**Figure 6:**
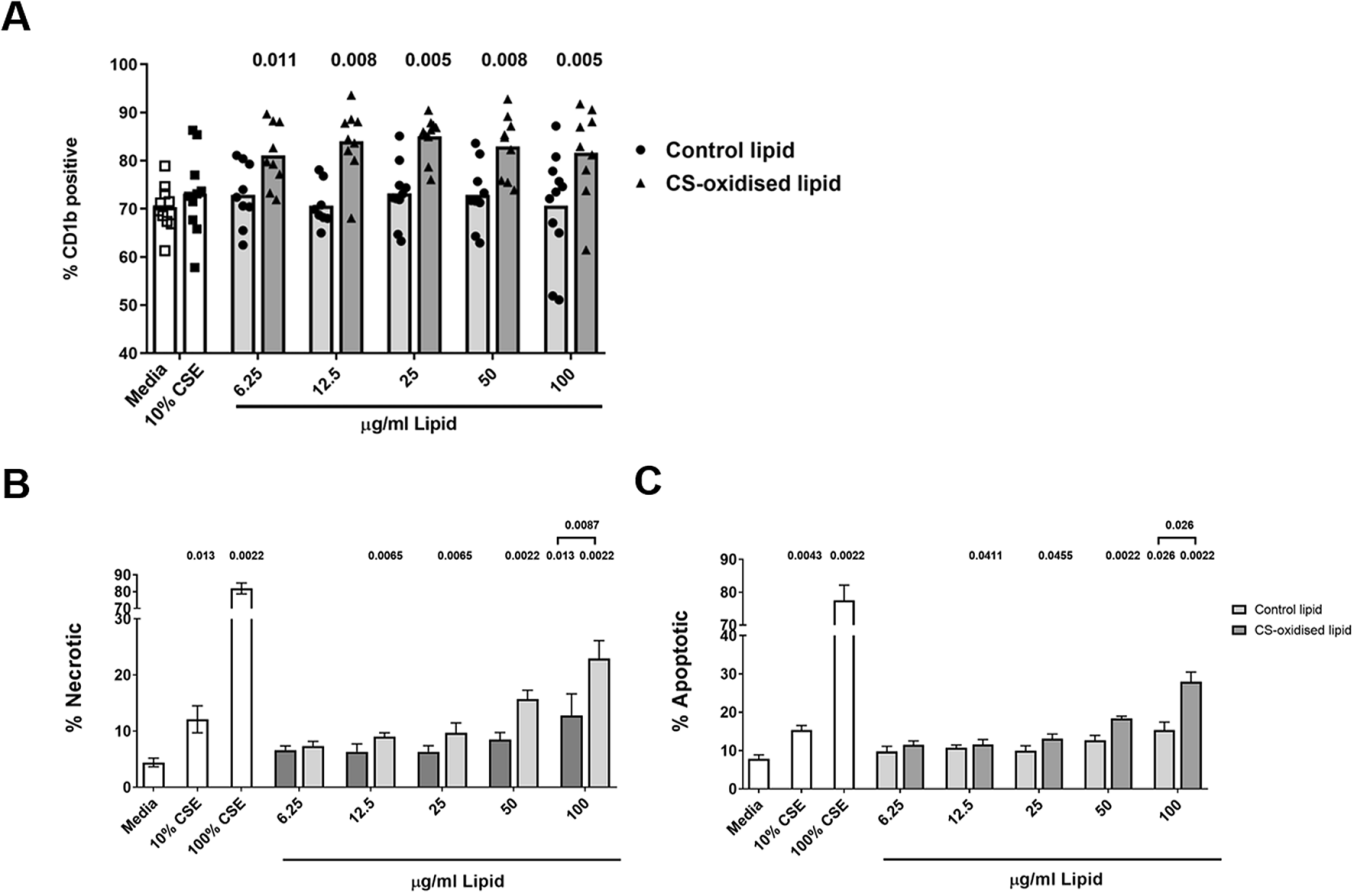
Oxidised epithelial cell lipids increase macrophage CD1b expression and cause epithelial cell death. (**A**) MDM or (**B**,**C**) 16HBE were exposed to media with or without cigarette smoke extract (CSE), with varying concentrations of CS-oxidised epithelial lipid (6.25–50 µg/mL) for 24 h, and CD1b expression was measured by flow cytometry using an unconjugated primary antibody and an APC-conjugated secondary antibody. *p* values vs control provided from Wilcoxon Signed Rank test. n = 9–10. Data are presented as mean with individual data points. Significance from control *p* < 0.05. (**B**) Necrosis of 16HBE cells measured by sytox positive cells (**C**) Apoptosis of 16HBE cells measured by Annexin V positive cells. Data represents mean ± SEM. n = 6, significance from control *p* < 0.05, Mann Whitney U test.

Exposure of MDM to CS-oxidised total cellular lipid at concentrations of 6.25–100 μg/mL significantly increased macrophage expression of CD1b (81.14–85.02% vs 70.3% for control) (Fig. 6). Treatment with control total cellular epithelial lipid did not significantly increase CD1b, nor did CSE.

### Oxidised epithelial cell lipids cause epithelial cell toxicity

Exposure of MDM to CS-oxidised total cellular lipid at concentrations of 12.5–100 μg/mL significantly increased bronchial epithelial necrosis (9–23% vs 4.4% for control) and apoptosis (11.6–27.9% vs 7.8% for control) (Fig. 6A). Treatment with control total cellular epithelial increased cell necrosis (12.9%) and apoptosis (15.4%) only at 100 μg/mL but significantly lower than CS-oxidised lipid at 100 μg/mL, and CSE showed dose dependent necrosis and apoptosis (Fig. 6B,C).

### Oxidised epithelial cell lipids inhibit macrophage phagocytic function

**Figure 7:**
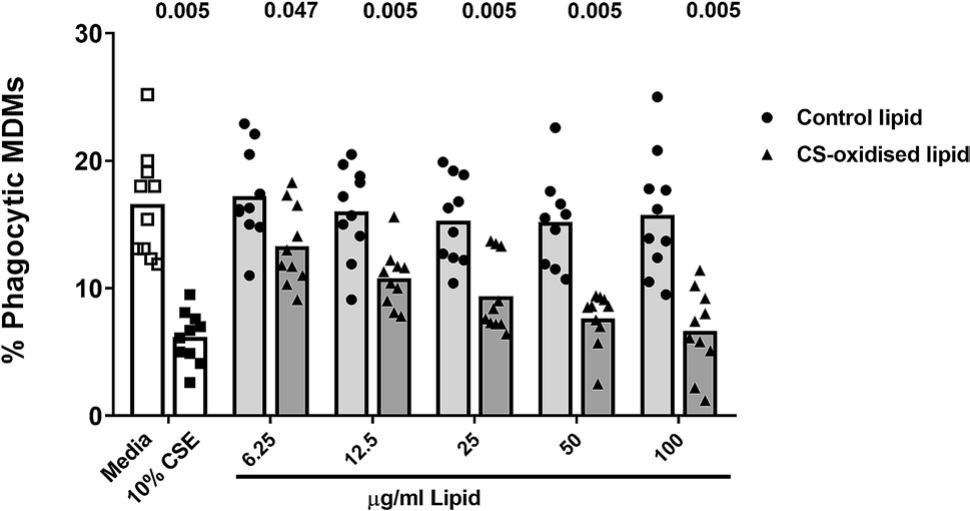
Oxidised epithelial cell lipids inhibit macrophage phagocytic function. MDM were exposed to media with or without cigarette smoke extract (CSE), with varying concentrations of CS-oxidised epithelial lipid (6.25–100 µg/mL) for 24 h, and phagocytosis was measured by flow cytometry. *p* values vs control provided from Wilcoxon Signed Rank test. n = 10. Data are presented as mean with individual data points.

Macrophage phagocytic function was suppressed in a dose dependent manner by the presence of oxidised epithelial lipid (6.66–13.31% vs 16.61% for control) (Fig. 7) but not in the presence of control lipid. The maximum inhibition of phagocytosis occurred in the presence of 100 µg/mL CS-oxidised lipid.

### Oxidised epithelial cell lipids suppress expression of mannose receptor

**Figure 8:**
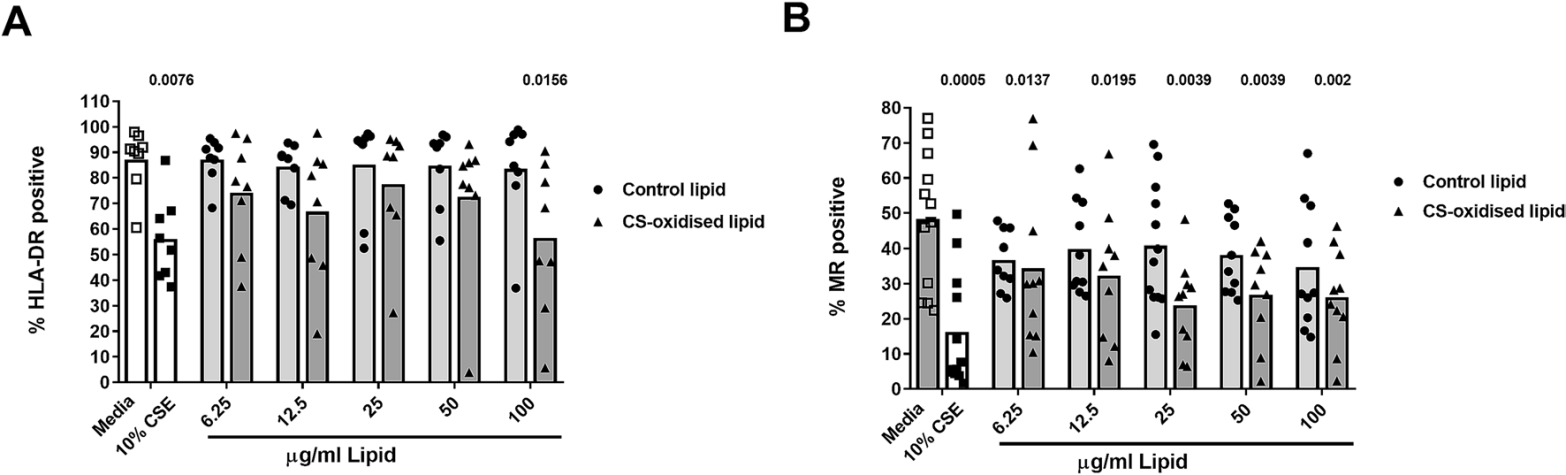
Oxidised epithelial cell lipids moderately suppress expression of mannose receptor and HLA-DR. MDM were exposed to media with or without cigarette smoke extract (CSE), with varying concentrations of CS-oxidised epithelial lipid (6.25–100 µg/mL) for 24 h, and (**A**) HLA-DR and (**B**) mannose receptor (MR) measured by flow cytometry. *p* values vs control provided from Wilcoxon Signed Rank test. n = 8. Data are presented as mean with individual data points.

There were no significant changes in the total expression of MHC class I antigen-presenting molecule (HLA-ABC) with any treatment (data not shown), and a small but significant decrease (56.45% vs 87.19% for control, *p* = 0.0156) in MHC class II (HLA-DR) only at the highest concentration of lipid tested (100 µg/mL, Fig. 8A). CSE similarly reduced MHC class II in line with our previous data (56.03%). However, cell surface expression of the macrophage phagocytic recognition molecule and lipid binding receptor, mannose receptor, was significantly reduced in the presence of CS-oxidised lipids (34.71–40.88% vs 48.28%, Fig. 8B) but not control lipids.

## Discussion

We introduce a new paradigm in COPD: that a self-maintaining process involving presentation of potentially immunogenic oxidised lipids via CD1b may be instigated but not necessarily perpetuated by cigarette smoking and prevents the normal resolution of the inflammatory response. The number of studies reporting self-antigens in COPD patients continues to rise in recent years, even in patients with mild disease and those who have ceased smoking. These include antinuclear antibodies in 34% of COPD patients at first exacerbation^2^, anti-elastin antibodies^3^ that correlate with emphysema severity, anti-epithelial cell antibodies^4^, IgG and C3 deposition in the small airways (indicative of antibody-mediated complement activation)^48^, neutrophil granule protein^5^, and a range of other autoantibodies^6^. Activation of effector T-cells specific for self-antigens can cause local inflammation by mechanisms that include increased production of pro-inflammatory cytokines by Th1 T-cells, increased proliferation of CD8+ cytotoxic T-cells, and cell injuries mediated by antigen–antibody complexes. We and others have shown an increased infiltration of activated CD8+ cytotoxic T-cells in COPD patients^49,50^, consistent with reports in autoimmune disease. An important study showed that Rag2−/− T-cell-deficient mice injected with T-cells from cigarette smoke-exposed mice develop COPD, indicating an immune response to self-antigens^51^.

We show that AM expression of CD1b was significantly increased in healthy smokers and both current- and ex-smokers with COPD as well as 8-isoprostane, a marker of lipid peroxidation, was also increased in the BAL and EBC of COPD patients regardless of current smoking status. Furthermore, potentially immunogenic oxidized lipids can be induced in bronchial epithelial cells by cigarette smoke exposure, and that these lipids can significantly upregulate the macrophage expression of CD1b, a molecule that is critical for binding and presenting lipid antigens to CD1b-restricted T-cells and binding by the TCR αβ and can activate CD1b restricted T-cells^34^. The lipids may also activate T cells by indirect effects involving induction of lipid autoantigens, CD1 transcription, or cytokine release^36^. The lack of increase on macrophages exposed to CSE but increase in the AM of smokers is further evidence that lipid in the airways is key to CD1b expression rather than an effect of the smoke itself, perhaps as part of the chronic inflammatory state in COPD airways induced by chronic smoking. Other studies have shown that oxidised LDL, often used to test the effects of oxidised lipid, can cause an increase in inflammatory cytokines in PBMCs^52^, lung epithelial cells^53^, and macrophages^54–56^. This inflammation in vivo may also lead, to antigen presenting cell activation^57^ and increased CD1b alongside the direct effects of the oxidised lipids observed on CD1b levels in this study. However, we observed that the classic MHC antigen-presenting molecules that do not present lipids were relatively unchanged in the presence of the oxidised lipids, with no significant changes in HLA class I and a significant decrease in class II only in the presence of the highest concentration of lipids tested. COPD AM have previously been shown to have reduced HLA-DR^58,59^, but our previous study showed that HLA-ABC was only reduced in currently smoking COPD patients and not ex-smokers^59^ further supporting the involvement of oxidised lipid in COPD. Little is known about CD1b due to its lack of expression in mice; however, it has been shown to recognise lipids including mycobacterial lipids and those exposed on apoptotic cells (e.g., phosphatidylserine and phosphatidylcholine) and present to small populations of CD1b restricted T cells^37,60,61^.

Our data showing increased sphingomyelin in the smoke-oxidised lipid, alongside the known role of Saposin-C in the CD1b pathway indicates a role for the sphingolipid pathway in COPD. This notion is in accordance with our and others data on a shift of sphingolipid pathway towards favouring inflammation in COPD/response to cigarette smoke, including ceramide overproduction, an upregulation of a range of sphingosine-1-phosphate (S1P) signalling-related genes including sphingosine kinases, but downregulation of their enzyme activity and subcellular redistribution in macrophages^62,63^. We also showed that a change in sphingosine pathway was likely involved in reduced efferocytosis and bacterial phagocytosis seen in COPD patients and macrophages exposed to cigarette smoke^39,40^. Hughes et al., found increased sphingomyelins in the BAL of smokers vs never-smokers^64^ and Telenga et al., showed that a range of sphingolipids, including sphingomyelins was increased in the sputum of current smoker COPD patients vs smokers and then reduced upon cessation of smoking in both groups^65^. The suggestion that the sphingosine pathway may be involved in an autoimmune component of COPD is further supported by the fact that modulation of the sphingosine pathway is in preclinical and clinical trials for a number autoimmune diseases already including rheumatoid arthritis^66^, psoriasis^67^, lupus^68^ and that a sphingolipid modulator is already FDA approved for treatment of multiple sclerosis^69^.

Our data also showed an increase in lysophosphotidylcholine in the total cellular lipid extracted from CS-exposed cells. This lipid has been shown to be exposed on the outer membrane of apoptotic cells much like phosophotidylcholine^15,70,71^. It is also associated with increased inflammation^72^ and increased apoptosis of various cell types which are exposed to it^72–76^ and may induce apoptosis an inflammation in airway epithelial cells, a hallmark of COPD patients^12,77^. Pasini et al. found elevated lysophosphotidylcholine in the plasma of smokers vs never-smokers and that exposure to oxidised lipids increased the level of lysophosphotidylcholine in never-smoker PBMCs^78^. Oxidised lipids are also known to induce apoptosis in cells^79,80^ and in this study we showed that CS-oxidised lipids were able to induce bronchial epithelial cell necrosis and apoptosis. Thus, the authors suggest that smoking results in increased lysophosphotidylcholine and oxidised lipids in the airways from apoptotic epithelial cells which in turn may trigger further apoptosis of cells in the airways as well as inflammation, contributing to a vicious cycle of ongoing airway cell apoptosis even after smoking may have ceased, something that is documented to occur in COPD ex-smoker patients^12,77^.

The phagocytosis data from this study suggests that the presence of epithelial cell-derived oxidised lipids in COPD could be one important cause of the impairment in macrophage capacity to phagocytose apoptotic bronchial epithelial cells that we and others have previously reported^9,10,81^. Many lipids present in the mitochondrial membrane of healthy cells are redistributed to the plasma membrane of apoptotic bodies and are effectively cleared by efferocytosis. If clearance is impaired, the exposed lipids could become oxidised by existing free radicals observed in the airways of COPD patients including ex-smoker COPD patients^18,19,81–84^. From there, lipid may be taken up into the cell by phagocytosis or direct fusion and endocytosis, be presented by immune cells such as alveolar macrophages, and recognised as ‘foreign’ by T cells. A resultant increased inflammatory effect has been shown in mice exposed to oxidised lipids^20,21^, antibodies to oxidised lipids were detected in cigarette smoke-exposed mice^21^ and in SLE, increased numbers of uncleared apoptotic cells resulting from defective efferocytosis induced anti-DNA antibodies directed against self-antigens (reviewed in^85^). Further reports showed that monoclonal autoantibodies specific for oxidised phospholipids can directly reduce macrophage efferocytic capacity^86,87^. Interestingly, we also showed that the oxidised lipids significantly downregulated macrophage expression of mannose receptor, an important phagocytic recognition molecule that we have previously shown to be decreased on AM from COPD subjects irrespective of their current smoking status^88^. In the context of the current study, it is also interesting to note that mannose receptor can co-localise with CD1b, delivering mycobacterial lipoglycan lipoarabinomannan (LAM) to late endosomes for loading onto CD1b^32^. Oxidised lipids are also likely to contribute to the inflammatory response in COPD; increased inflammation in mice exposed to oxidised lipids has been shown^20,21^, while systemic oxidative stress caused by cigarette smoking also increased levels of oxidised phospholipids in peripheral blood monocytes of smokers^87^.

## Conclusion

In summary, our new findings provide a possible new pathway for why COPD disease continues to progress despite cessation of smoking and a possible cause for the hallmark AM phagocytic dysfunction in COPD. Identification of new interventions that modulate CD1b lipid presentation, perhaps via modulation of the sphingosine pathway, will have relevance in COPD and other chronic inflammatory lung diseases including Idiopathic Pulmonary Fibrosis and asthma, where the presence of an autoimmune component has also been proposed.

## Supplementary Information

Supplementary Information.

## Data Availability

All data generated or analysed during this study are included in this published article (and its “Supplementary Information S1” files).

## Abbreviations

16HBE: 16HBE14o-bronchial epithelial cell line
AM: Alveolar macrophage
AP: Adaptor protein
BAL: Bronchoalveolar lavage
BALF: Bronchoalveolar lavage fluid, cell free
C3: Complement component C3
COPD: Chronic Obstructive Pulmonary Disease
CS: Cigarette smoke
CSE: Cigarette smoke extract
CSH: Charged surface hybrid
EBC: Exhaled breath condensate
EIC: Extracted ion chromatograms
FDA: Food and Drug Administration
FEV: Forced expiratory volume
GOLD: Global Initiative for Chronic Obstructive Lung Disease
HBSS: Hanks’ balanced salt solution
HLA: Human leukocyte antigen
MDM: Monocyte derived macrophage
MeCN:IPA: Acetonitrile:isopropanol
MFI: Mena fluorescent intensity
MHC: Major histocompatibility complex
MR: Mannose receptor
NTHi: Non-typeable *Haemophilus influenzae*
PBMC: Peripheral blood mononuclear cell
QC: Quality control
RT: Retention time
S1P: Sphingosine-1-phosphate
SEM: Standard error of the mean
SLE: Systemic lupus erythematosus
TCR: T cell receptor
UPLC: Ultra performance liquid chromatography
