# Supplementary material for: The role of oxidised self-lipids and alveolar macrophage CD1b expression in COPD
Source: Sci Rep. 2021 Feb 18;11:4106. doi: 10.1038/s41598-021-82481-0 (PMC7892841; doi:10.1038/s41598-021-82481-0)
Supplement: Supplementary file 1 — Supplementary Information. [file 41598_2021_82481_MOESM1_ESM.docx]

The role of oxidised self-lipids and alveolar macrophage CD1b expression in COPD

Miranda Ween^1,2*^, Jake B White^2,3^, Hai Tran^1,2^, Violet Mukaro^1,5^, Charles Jones^2^, Matthew Macowan^1,2^, Greg Hodge^1,2^, Paul J Trim^2,3^, Marten F Snel^2,3^, Sandra J Hodge^1,2^

^1^Department of Thoracic Medicine, Royal Adelaide Hospital, Australia

^2^School of Medicine, Faculty of Health Sciences, University of Adelaide, Australia

^3^Proteomics, Metabolomics and MS Imaging Core Facility, South Australian Health and Medical Research Institute (SAHMRI), Australia.

4. Vascular Research Centre, Lifelong Health Theme, South Australian Health and Medical Research Institute (SAHMRI), Australia.

5. Centre for Integrated Critical Care, University of Melbourne, Melbourne, Victoria

**Supplemental Methods**

***Monocyte isolation and generation of M2 monocyte derived macrophages***

PBMCs were generated as per the protocol in the main article. Monocytes were purified by adhesion to plastic for 1hr then assessed for CD1b expression. M2-like MDMs were differentiated by exposure of monocytes to RPMI 10% FCS and 100 ng/mL M-CSF for 12 days, with media changed on day 4 and day 8 and assessed for CD1b expression. CD1b expression was assessed by flow cytometry using a mouse-anti-CD1b primary antibody and an APC-conjugated secondary antibody and positivity gated based on a secondary antibody only control.


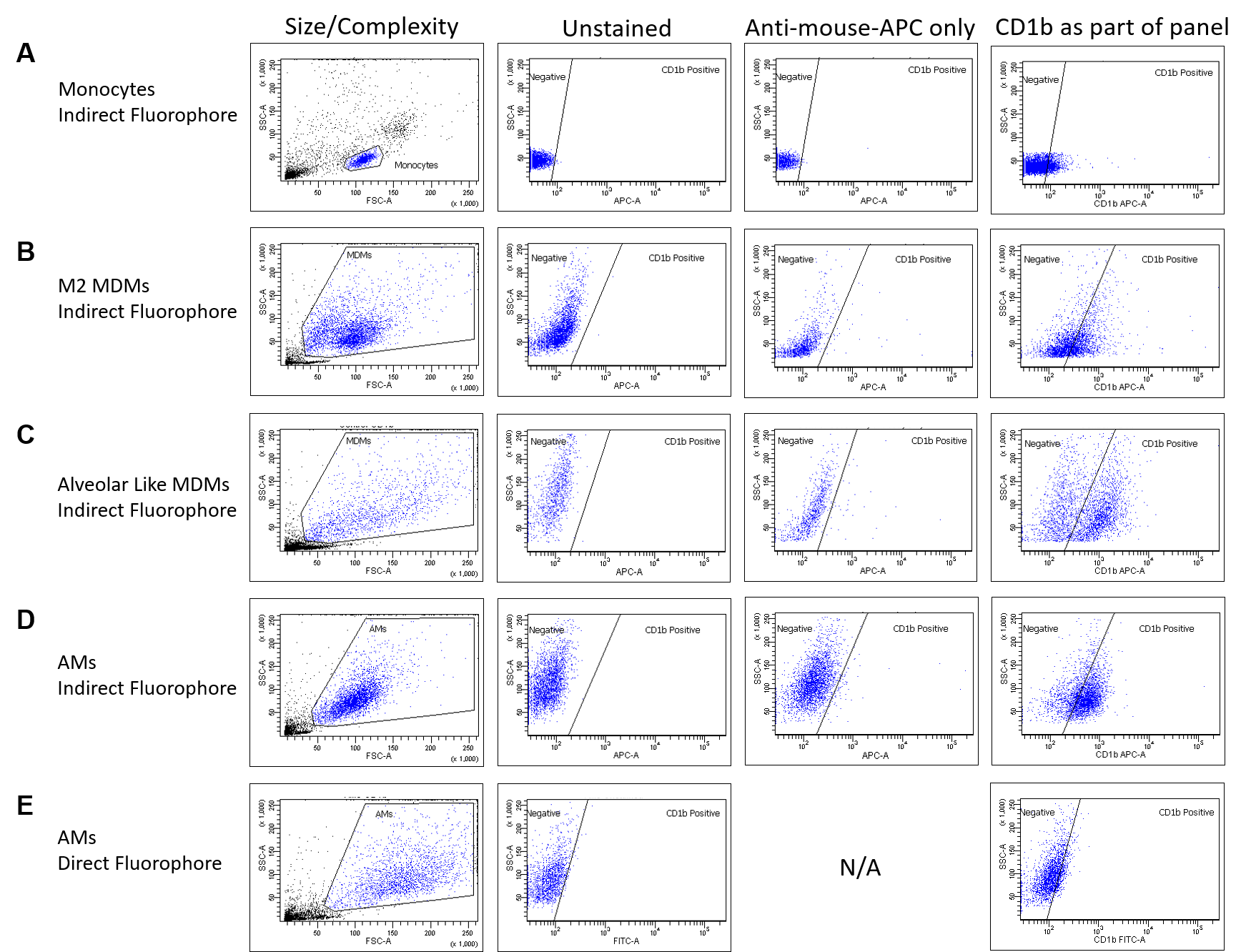


**Figure S1. Representative flow cytometry dot plots showing CD1b staining in cells from healthy donors.**

Images represent indirect staining utilising a Mouse-anti-CD1b primary antibody and an APC-conjugated secondary antibody of (A) Monocytes (B) Alveolar like M1 MDM (C) M2 MDM (D) AM and (E) Direct labelling of AM with the FITC conjugated anti-CD1b antibody used in Figure 2. Final panels on the right represent images from Monocyte staining panel: HLA-DR-FITC/CD1b-APC, M1 MDM panel: HLA-ABC-FITC/CD1b-APC, M2 MDM panel: CD206-FITC/CD1b-APC, AM panel CD1b-FITC/CD31-PE/CD14-PerCP.


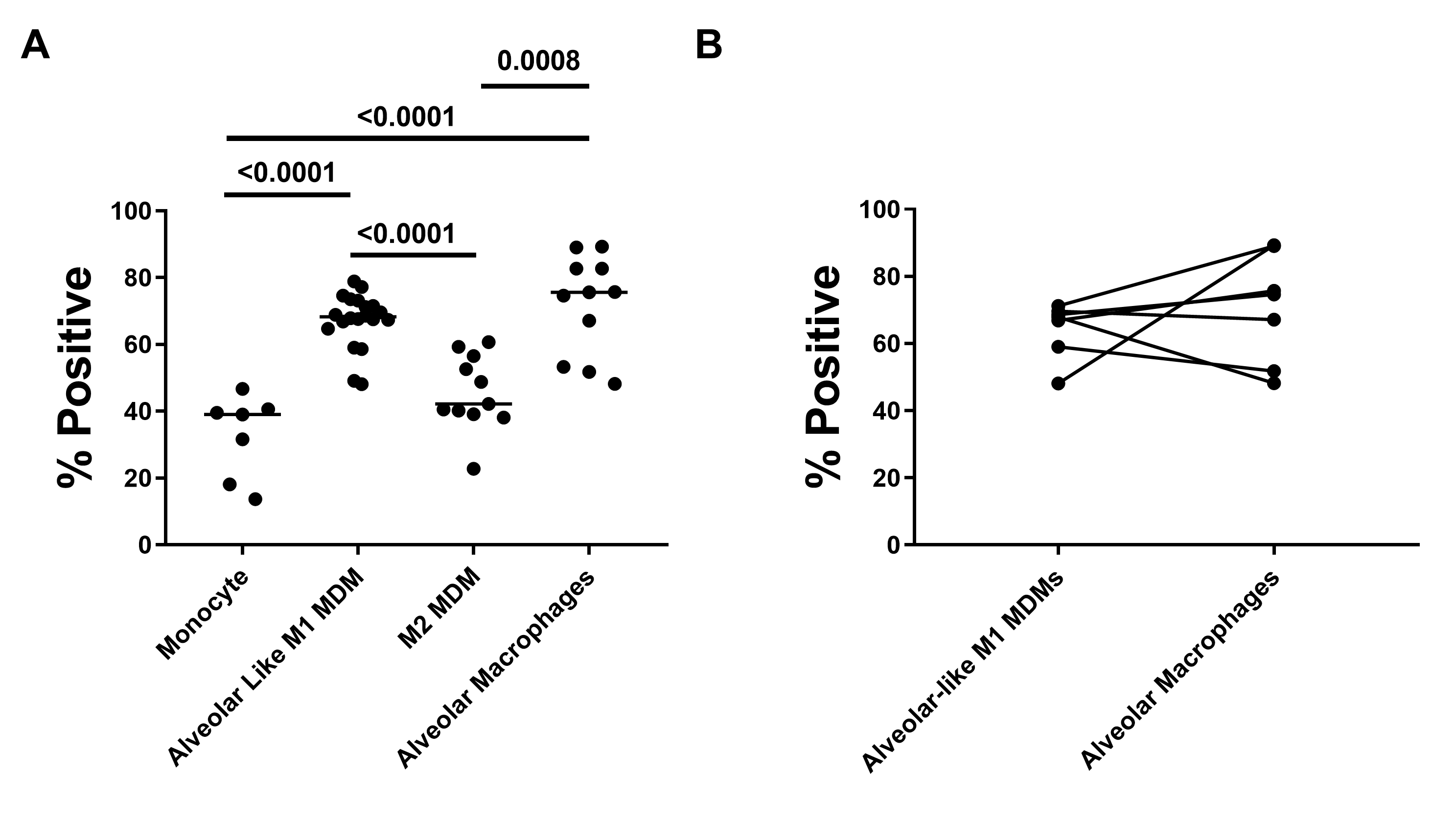


**Figure S2. CD1b expression in healthy donors.**

Cells were labelled with mouse-anti-CD1b antibody and APC-conjugated secondary antibody. (A) Comparison of cell types (B) matched samples from the same donor. Monocyte staining panel: HLA-DR-FITC/CD1b-APC, M1 MDM panel: HLA-ABC-FITC/CD1b-APC, M2 MDM panel: CD206-FITC/CD1b-APC, AM panel HLA-ABC-FITC/CD1b-APC.


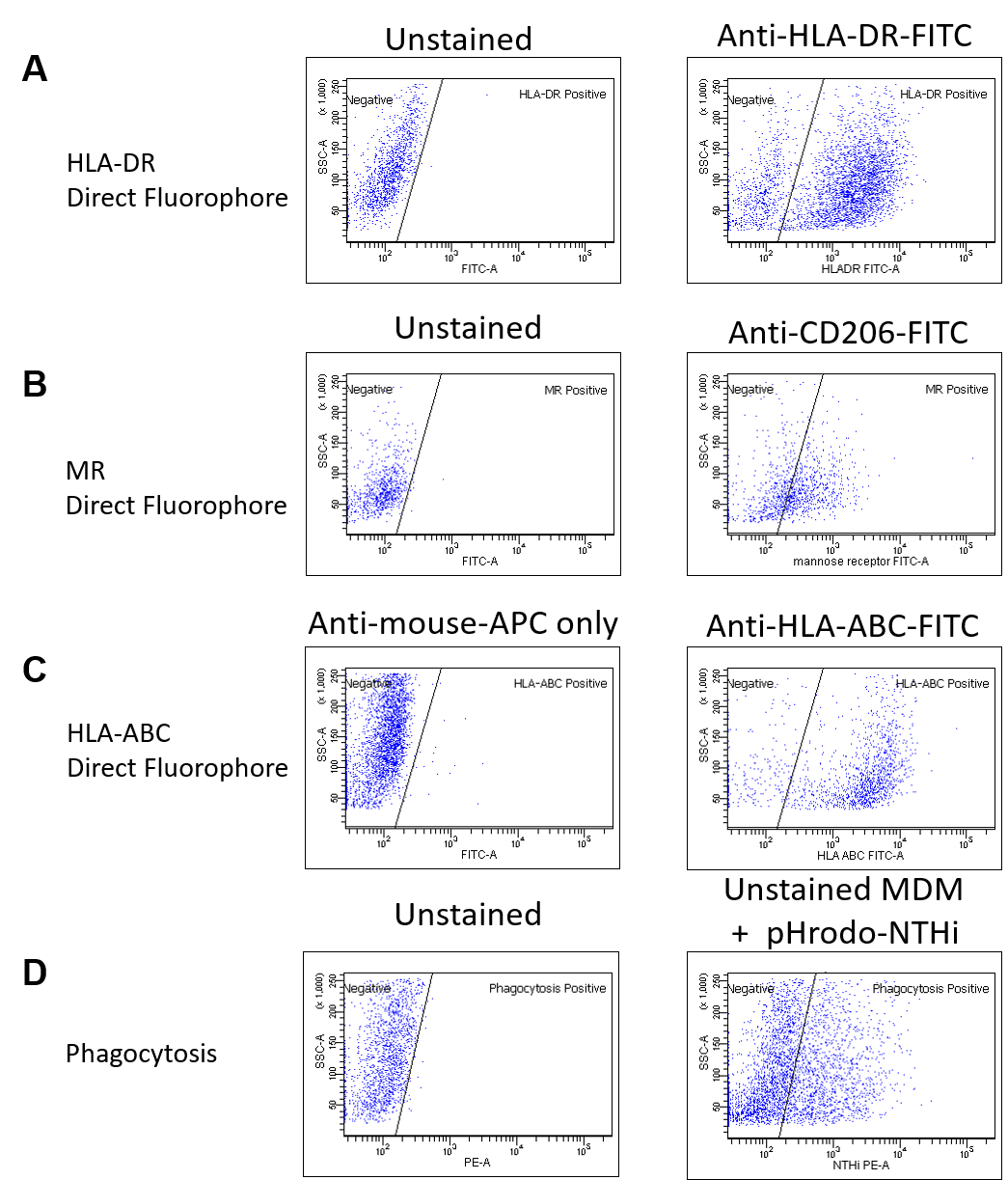


**Figure S3. Representative flow cytometry dot plots showing from healthy donors.**

M1 alveolar-like MDMs direct fluorophore labelling to detect (A) HLA-DR (B) Mannose Receptor (C) HLA-ABC or detection of (D) MDMs which have phagocytosed pHrodo red labelled NTHi. M1 MDM panels: (1) HLA-ABC-FITC/CD1b-APC (2) HLA-DR-FITC (3) CD206-FITC (mannose receptor). Phagocytosis assays only utilised a single pHrodo stain for the bacteria, the macrophages were not stained in any way. Bacteria alone fall outside the macrophage gate due to their small size.


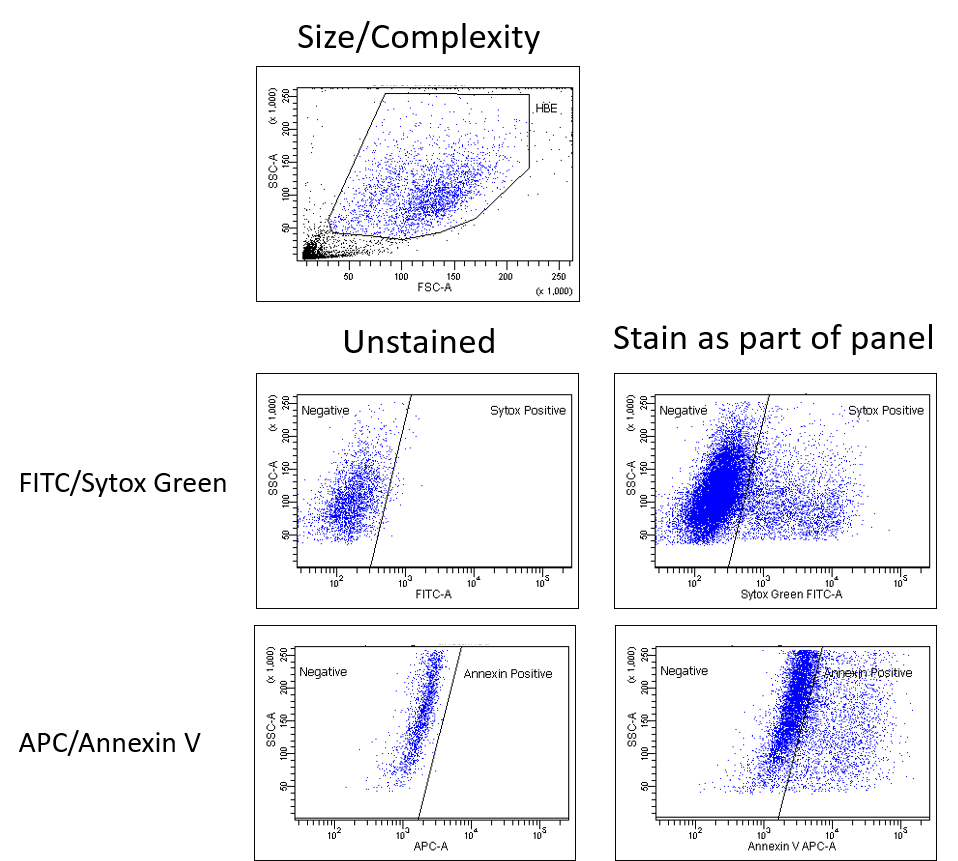


**Figure S4. Representative flow cytometry dot plots showing Annexin and Sytox staining in 16HBE cells.**

16HBE Cells were dual stained with Sytox Green as a necrosis marker and Annexin-V-APC as an apoptosis marker.
